# A predictive model based on site-specific risk factors of recurrence regions in endometrial cancer patients

**DOI:** 10.1186/s12885-022-10193-3

**Published:** 2022-10-31

**Authors:** Wonkyo Shin, Seong J. Yang, Sang-Yoon Park, Sokbom Kang, Dong Ock Lee, Myong Cheol Lim, Sang-Soo Seo

**Affiliations:** 1 Center for Gynecologic Cancer, National Cancer Center, Goyang, Republic of Korea; 2 Department of Obstetrics & Gynecology, Chungnam National University Sejong Hospital, Sejong, Republic of Korea; 3 Department of Statistics (Institute of Applied Statistics), Jeonbuk National University, Jeonju, Republic of Korea; 4 National Cancer Center, 323 Ilsan-ro, Ilsandong-gu, 10408 Goyang-si Gyeonggi-do, Republic of Korea

**Keywords:** Endometrial cancer, Lymph node, Lymph node dissection, Survival rate

## Abstract

**Objective:**

This study investigated site-specific differences in clinical factors for recurrence in patients who were newly diagnosed and treated for endometrial cancer. A model for predicting recurrence sites was generated.

**Methods:**

Electronic medical records’ data were retrieved from January 2006 to December 2018 for patients who were diagnosed with endometrial cancer at the National cancer center in Korea. Recurrence sites were classified as local, regional, or distant. We used multinomial logistic regression models that modeled the log-odds for the three recurrence sites relative to non-recurrence as a linear combination of possible risk factors for the recurrence of endometrial cancer.

**Results:**

The data of 611 patients were selected for analysis; there were 20, 12, and 25 cases of local, regional, and distant recurrence, respectively, and 554 patients had no recurrence. High-grade disease was associated with local recurrence; non-endometrioid histology and parametrial invasion were risk factors for regional recurrence; additionally, parametrial invasion and no lymphadenectomy were associated with distant metastasis.

**Conclusion:**

We identified different risk factors specific for each type of recurrence site. Using these risk factors, we suggest that individually tailored adjuvant treatments be introduced for patients.

## Introduction

Endometrial cancer is usually detected in patients at an early stage. Specific symptoms include vaginal discharge and abnormal vaginal bleeding [1]. If detected in the early stage, the five-year survival rate after surgical treatment has a prognosis of more than 90% [2, 3]. Even in the advanced stage, it has a modest survival rate and can be managed through surgery and adjuvant chemotherapy or radiation therapy. However, the treatment prognosis is poor in cases with recurrence. The diagnosis of endometrial cancer is confirmed through biopsy of the endometrium, which is followed by surgical treatment and additional treatment that is determined according to the stage identified in the pathology report.

In general, cancer recurrence can be classified broadly into local, regional, and distant recurrence [2]. However, identifying risk factors for recurrence remains difficult in gynecologic oncology. Widely known risk factors for recurrence include myometrial invasion depth, lymphovascular invasion, cervical stromal invasion, parametrial invasion, and lymph node metastasis [4].

The best treatment option for adjuvant treatment among concurrent chemo-radiotherapy (CCRT) and the addition of systemic chemotherapy, radiotherapy, or use of the sandwich method (alternating chemotherapy and radiation therapy), is controversial [5–7]. However, as the addition of adjuvant treatment increases, the patient’s quality of life can worsen, and morbidity could increase.

Therefore, if the risk factors are different depending on the recurrence pattern, the recurrence rate might be lowered through appropriately tailored adjuvant treatment. Therefore, we attempted to identify risk factors for each recurrence type and analyze them using multinomial logistic regression analysis.

## Methods

We reviewed the electronic medical records of patients who were newly diagnosed with endometrial cancer using endometrial biopsy and treated at the National Cancer Center in South Korea between January 2006 and December 2018. Patients were eligible if diagnosed with endometrial cancer by pathologic reports, ages 18 to 80. Patients with other co-existing cancers, > 3 points of Eastern Cooperative Oncology Group performance score, severe cardiovascular disease, or respiratory disease were excluded. This retrospective study was approved by the Institutional Review Board of our institution (IRB No. NCC2019-0272), and the requirement for informed consent was waived due to the retrospective nature of the study.

We collected the data on clinical factors for 611 patients, including information on age at diagnosis, the International Federation of Gynecology and Obstetrics (FIGO) stage, FIGO grade, histology of the surgically removed tissues, surgical approach, radicality of hysterectomy, lymph node dissection (LND), lymphovascular invasion, and the history of adjuvant chemotherapy or radiotherapy. Staging surgery was performed in all patients, and adjuvant chemotherapy or radiation therapy was performed based on the surgical outcomes. For chemotherapy, adriamycin-cisplatin was used before 2015, and the regimen was changed to paclitaxel-carboplatin. Radiation therapy was administered based on the pelvic and/or para-aortic pathologic reports or imaging results, extra-beam radiation treatment (EBRT), and intracavitory radiation (ICR) were added in some cases by risk factors according to pathologic reports; however, it was not accurately recorded. Risk group sub-categorization was performed. An endometrioid histology of FIGO grade 1 or 2 and confined to the endometrium or with < 50% myometrial invasion was defined as low risk; stage IA grade 3 and stage IB grade 1 or 2 were defined as intermediate risk; stage IB grade 3 or stages II, III, or IV disease were defined as high risk and any stage of non-endometrioid histology disease was defined as high risk [8]. These clinical risk factors were classified using the European Society for Medical Oncology guidelines [9]. Recurrence was confirmed through imaging tests according to the follow-up period after treatment. Local recurrence was defined as vaginal stump or under pelvic brim region recurrence, regional recurrence was pelvic or para-aortic lymph node metastasis or intra-abdominal upper pelvic brim recurrence, and distant metastasis was another organ metastasis and or extra-abdominal metastases. During the first 2 years, serum tumor marker was checked between 3 months, CT scan was performed in 6 months, from 2 to 5 years, 6 months blood lab check, yearly imaging follow up. Therefore, even if there were several recurrence sites, it could be inaccurate. Moreover, recurrence sites were classified into three categories, and three patients had overlapping relapses. In this case, priority was given in the order of local, regional, and distant recurrence to assign patients to a higher priority group. *P*-values < 0.05 were considered statistically significant.

We noted that our tests’ familywise Type-I error rate (FWTE) exceeds 0.05. The Bonferroni correction guarantees that FWTE is < 0.05, but this correction may be too conservative with dependent multiple tests as our case and results in reducing the power of tests. Since this study focused on finding site-specific clinical factors for recurrence, it is important to identify potentially significant factors. In this sense, we did not consider a multiple testing correction.

The data were analyzed using the logistic regression model, which can be used to model a categorical response. The response variable was grouped into four levels as follows: non-recurrence, local recurrence, regional recurrence, or distant recurrence. A univariate logistic regression model was used to evaluate the individual effect of each risk factor. A multivariate logistic regression model was also fitted for a comprehensive analysis and understanding. Since the response variable of interest was not binary and had multiple levels, the multinomial logistic regression model was employed. This model is a direct extension of the conventional binary logistic regression model because the response variable has levels greater than three. [10] Using this model enabled an assessment of the log-odds for the recurrence of endometrial cancer at the three recurrence sites relative to non-recurrence as well as the estimation of the conditional probability for recurrence based on the risk factors for each site (or non-recurrence). For additional comparison, the three recurrence sites were merged into one group and fitted to a binary logistic regression model.

**Fig. 1:**
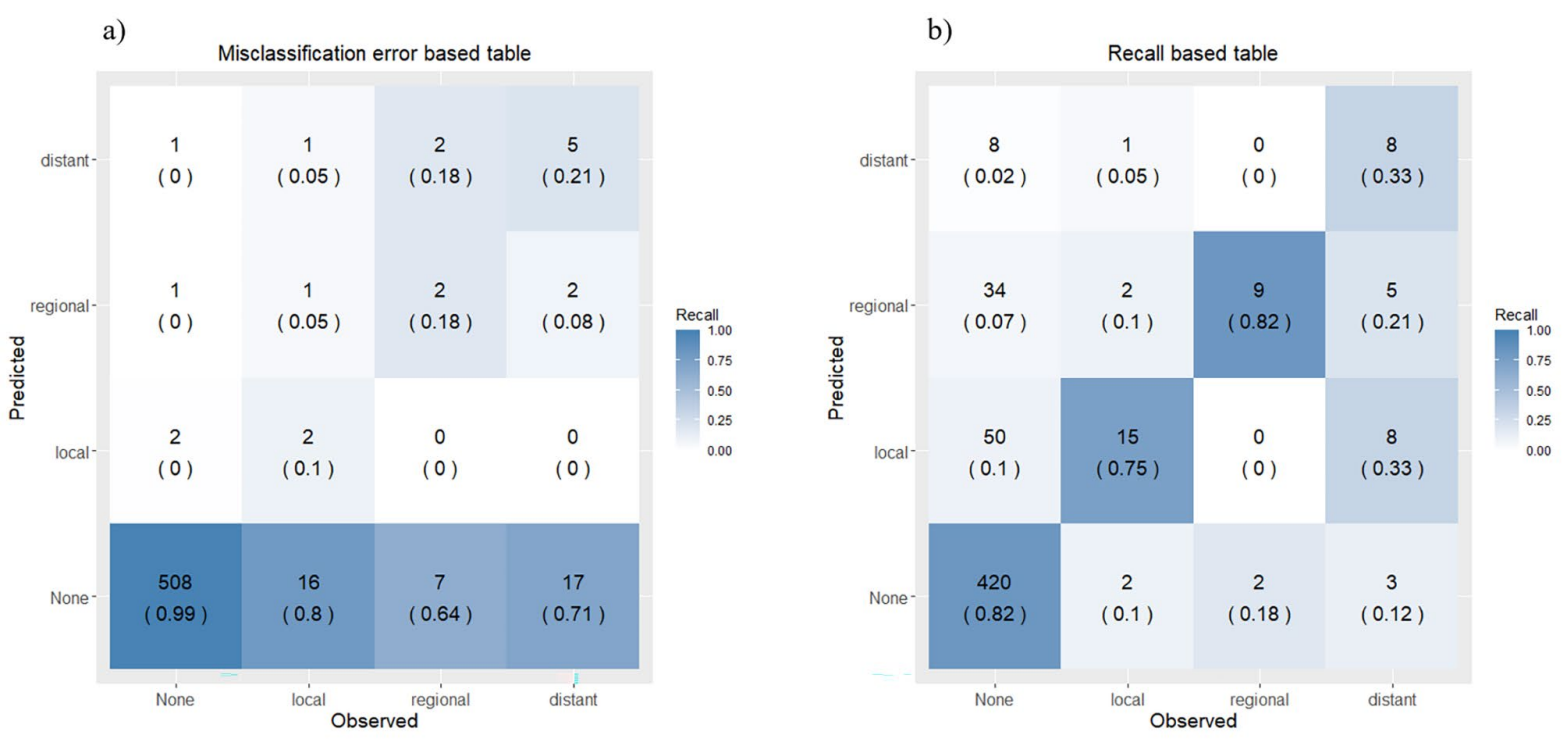
Comparisons of the fitted model versus the observed data, a) original model b) modified model for focusing recall measure. The number displayed on each cell represent the number of patients belonging to the cell and the corresponding recall value (in parentheses), respectively.

Subsequently, we produced 4 × 4 tables to summarize the comparisons of the fitted model versus the observed data. The rows and columns of the tables represent the instances for the actual and predicted levels by the fitted model. The tables were visualized as heat maps, which are presented in Fig. 1. We noted that by default, each observation was classified into the level that maximized the estimated conditional probability. This is known to minimize the overall misclassification error rate, which is the proportion of the misclassified observations out of the total sample, and tends to emphasize the majority class. This might have resulted in a poor prediction for patients with recurrence since the majority of our data were for non-recurrence, and the data were quite imbalanced.

To address this issue, we modified our initial prediction by adjusting the estimated conditional probability using different weighting factors. This produced various 4 × 4 tables, and we calculated a specific performance measure different from the misclassification error for each table. We focused on the *recall* measure, which refers to the proportion of patients that were classified correctly out of the actual number since it may be important that as many relapse patients as possible are correctly detected. Generally, recall is also known as the sensitivity and specificity in binary classification. We obtained the average value of the recall for the four levels of each table and subsequently selected the best one that gave the maximum average recall. This modified table is depicted in Fig. 1 along with the initial table, which indicated the minimized misclassification error.

## Results

Of the 611 patients selected for analysis, 20, 12, and 25 had local, regional, and distant recurrence, respectively. Table 1 summarizes the baseline characteristics of the patients for each recurrence site and for the 554 patients with no recurrence. The patients with recurrence were relatively older than those with no recurrence. The rates of non-endometrioid histology, lymphovascular invasion, and parametrial invasion were higher in patients with recurrence than in those with no recurrence, regardless of the site of recurrence. The recurrence rate in the high-risk group was higher than that in other groups. Lymph node metastasis and history of adjuvant treatment were observed more frequently in patients with recurrence than in those without recurrence. More patients with distant recurrence tended to have higher FIGO stages, and there was no significant difference in the FIGO grade.

**Table 1:** Baseline characteristics of patients

	value	No recur (N = 554)		Local (N = 20)		Regional (N = 12)		Distant (N = 25)	
		No.	Percent (%)	No.	Percent (%)	No.	Percent (%)	No.	Percent (%)
Age at diagnosis(continuous)		52.80 ± 10.28		58.00 ± 9.90		60.83 ± 11.21		55.68 ± 11.89	
Tumor size(continuous)	size(cm)	2.62 ± 2.32		4.07 ± 2.55		4.13 ± 3.60		4.72 ± 3.40	
Histology	endometrioid	467	0.84	11	0.55	7	0.58	11	0.44
	non-Endometrioid	87	0.16	9	0.45	5	0.42	14	0.56
FIGO stage	I II	492	0.89	16	0.80	6	0.50	9	0.36
	III IV	62	0.11	4	0.20	6	0.50	16	0.64
FIGO grade	1	280	0.51	6	0.30	2	0.17	2	0.08
	2	150	0.27	9	0.45	3	0.25	8	0.32
	3	70	0.13	0	0.00	2	0.17	4	0.16
	N/A	54	0.10	5	0.25	5	0.42	11	0.44
Surgical approach	Laparoscopy	392	0.71	12	0.60	4	0.33	7	0.28
	Laparotomy	162	0.29	8	0.40	8	0.67	18	0.72
Pelvic LN metastasis	Negative	418	0.75	13	0.65	6	0.50	11	0.44
	Positive	37	0.07	1	0.05	3	0.25	12	0.48
	Not done	99	0.18	6	0.30	3	0.25	2	0.08
Paraaortic LN metastasis	Negative	309	0.56	10	0.50	3	0.25	12	0.48
	Positive	27	0.05	2	0.10	2	0.17	11	0.44
	Not done	218	0.39	8	0.40	7	0.58	2	0.08
LVSI	No	454	0.82	12	0.60	7	0.58	10	0.40
	Yes	100	0.18	8	0.40	5	0.42	15	0.60
Parametrial invasion	No	552	1.00	19	0.95	9	0.75	18	0.72
	Yes	2	0.00	1	0.05	3	0.25	7	0.28
Risk group	Low	315	0.57	6	0.30	2	0.17	4	0.16
	Medium	87	0.16	4	0.20	3	0.25	2	0.08
	High	152	0.27	10	0.50	7	0.58	19	0.76
Adjuvant Chemotherapy	No	397	0.72	2	0.10	0	0.00	0	0.00
	Yes	157	0.28	18	0.90	12	1.00	25	1.00
Adjuvant Radiotherapy	No	438	0.79	7	0.35	6	0.50	10	0.40
	Yes	116	0.21	13	0.65	6	0.50	15	0.60

Each risk factor was evaluated for its association with recurrence using the binomial logistic regression model. Univariate models were used to assess individual effects of the risk factors; however, some data were excluded because of missing values so that the model could not be fitted. Pelvic or para-aortic lymph node metastasis, non-endometrioid histology, LVSI, a high FIGO grade, high FIGO stage, parametrial invasion, myometrial invasion depth, prior adjuvant treatment (chemotherapy or radiotherapy), age and large tumor size were related to recurrence (Table 2).

**Table 2:** Univariable analysis of risk factors

Variable	value	Binomial logistic model			
		Recurrence			
		RR(Risk Ratio) *	95% CI		p
Surgical approach	Laparoscopy	reference level			
	Laparotomy	3.577	2.055	6.331	**< 0.001**
Pelvic LN	Negative	reference level			
	Positive	6.025	2.967	11.974	**< 0.001**
	Not done	1.548	0.720	3.111	0.237
Paraaortic LN	Negative	reference level			
	Positive	6.867	3.203	14.523	**< 0.001**
	Not done	0.964	0.500	1.816	0.910
LVSI	No	reference level			
	Yes	4.383	2.492	7.712	**< 0.001**
Histology	Endometrioid	reference level			
	Non-endometrioid	5.183	2.932	9.165	**< 0.001**
FIGO grade	1	reference level			
	2	3.733	1.741	8.510	**< 0.001**
	3	2.400	0.793	6.690	0.101
	N/A	10.889	4.970	25.378	**< 0.001**
Parametrial invasion	No	reference level			
	Yes	66.000	17.082	> 100	**< 0.001**
FIGO stage	I, II	reference level			
	III, IV	6.656	3.697	11.949	**< 0.001**
Risk group	1	reference level			
	2	2.716	1.077	6.626	**0.029**
	3	6.217	3.233	12.779	**< 0.001**
Chemotherapy	No	reference level			
	Yes	69.538	21.321	> 100	**< 0.001**
Radiotherapy	No	reference level			
	Yes	5.582	3.184	9.953	**0.005**
Age	age	1.045	1.018	1.073	**0.001**
Tumor size	Size (cm)	1.270	1.153	1.399	**< 0.001**
Myometrial invasion		15.318	6.583	37.191	**< 0.001**

* ratio of the probability of recurrence to the probability of non-recurrence

Next, we analyzed the risk factors according to each reference site (Table 3), using multivariate logistic regression models. Among the clinical factors, ‘risk group’ and ‘prior radiotherapy’ were excluded in the multivariate model analysis. Risk group is a factor made up of a combination of other risk factors, while prior radiotherapy, is a factor that determines whether radiotherapy is performed after surgery, and it was removed because the available records were not enough. In the local recurrence group, a high FIGO grade, parametrial invasion, previous chemotherapy, and myometrial invasion depths were associated with recurrence. In the regional recurrence group, no para-aortic lymphadenectomy, non-endometrioid histology, parametrial invasion, and history of chemotherapy and age were associated with recurrence. In the distant metastasis group, no lymphadenectomy, the parametrial invasion, and prior chemotherapy had an effect on recurrence.

**Table 3:** Multinomial logistic regression analysis of clinical factors that related to site specific recurrence

Variables	value	No.	binomial logistic model				multinomial logistic model											
			Total recurrene (N = 57)				Local recurrence (N = 20)				Regional recurrence (N = 12)				Distant recurrence (N = 25)			
			risk ratio	95% CI		p	risk ratio	95% CI		p	risk ratio	95% CI		p	risk ratio	95% CI		p
Surgical approach	Laparoscopy	392	reference level															
	Laparotomy	162	0.788	0.339	1.808	0.575	0.681	0.198	2.342	0.542	1.164	0.182	7.455	0.873	0.902	0.267	3.045	0.868
Pelvic LN	negative	418	reference level															
	positive	37	0.362	0.083	1.433	0.157	0.039	0.001	1.926	0.103	2.380	0.112	50.632	0.578	0.425	0.076	2.363	0.328
	not done	99	2.003	0.559	7.733	0.295	3.642	0.557	23.820	0.177	0.424	0.046	3.871	0.447	> 100	> 100	> 100	< 0.001
paraaortic LN	Negative	309	reference level															
	Positive	27	1.912	0.500	7.923	0.352	3.051	0.267	34.840	0.369	1.740	0.114	26.460	0.690	2.473	0.479	12.760	0.279
	not done	218	1.023	0.313	3.007	0.968	0.741	0.133	4.126	0.732	14.820	1.407	156.100	0.025	0.000	0.000	0.000	< 0.001
LVSI	No	454	reference level															
	Yes	100	1.954	0.788	4.847	0.146	2.357	0.676	8.210	0.178	3.494	0.481	25.393	0.216	1.594	0.417	6.098	0.496
Histology	Endometrioid	467	reference level															
	Non-endometrioid	87	0.602	0.160	1.962	0.422	1.514	0.333	6.890	0.591	0.000	0.000	0.000	< 0.001	0.292	0.035	2.453	0.257
FIGO grade	1	280	reference level															
	2	150	1.335	0.479	3.837	0.583	0.945	0.252	3.546	0.934	1.384	0.093	20.683	0.814	2.187	0.358	13.377	0.397
	3	70	0.264	0.049	1.142	0.091	0.000	0.000	0.000	< 0.001	2.044	0.108	38.800	0.634	0.450	0.047	4.346	0.490
	N/A	54	1.418	0.308	7.162	0.661	0.323	0.043	2.396	0.269	> 100	> 100	> 100	< 0.001	4.970	0.334	74.020	0.245
Parametrial invasion	No	552	reference level															
	Yes	2	34.183	6.010	305.46	< 0.001	109.94	1.595	7576.60	0.030	68.300	3.865	1206.90	0.004	36.040	3.984	325.900	0.001
FIGO stage	I II	492	reference level															
	III IV	62	0.548	0.164	1.651	0.303	0.280	0.041	1.896	0.192	0.669	0.078	5.732	0.714	0.536	0.102	2.834	0.463
Chemotherapy	No	397	reference level															
	Yes	157	67.174	17.750	447.497	< 0.001	36.500	6.716	198.400	< 0.001	> 100	> 100	> 100	< 0.001	> 100	> 100	> 100	< 0.001
Age (Continuous)	age	-	1.037	0.998	1.079	0.068	1.032	0.976	1.091	0.269	1.110	1.022	1.206	0.013	1.002	0.945	1.061	0.959
tumor size (continuous)	size	-	1.041	0.896	1.208	0.593	1.083	0.852	1.378	0.514	1.143	0.857	1.525	0.362	1.023	0.846	1.236	0.817
Myometrial invasion		-	4.911	1.205	20.655	0.027	10.605	1.565	71.868	0.016	0.115	0.003	3.963	0.231	7.446	0.890	62.262	0.064

Based on these results, a model was created to predict the group with the greatest recurrence probability according to the risk factors in the patients (Fig. 1). The overall prediction accuracy was 91%. For patients with recurrence, the accuracy (recall) for each site was 10%, 18%, and 21% for local, regional, and distant recurrence, respectively. Therefore, the prediction for patients with recurrence was poor compared to the overall prediction performance (this is shown in the left panel of Fig. 1). Here, the shades of the color represent the recall value for each class. The frequency and recall (in parentheses) of the tables were also labeled as text. The right panel of Fig. 1 shows the results of modifying our initial prediction by focusing on the recall. Although the overall misclassification error declined from 91 to 80%, there were drastic gains in the recall as follows: 10%–>75% (local), 18%–>82% (regional), and 21%–>33% (distant).

## Discussion

To the best of our knowledge, this is the first study to analyze the relationship between the risk factors for endometrial cancer and the recurrence sites. Endometrial cancer is diagnosed relatively early, and a cure can be expected with a relatively small range of surgery in the early stage. When premenopausal women are diagnosed, many fertility saving approaches can be adopted. First, there have been studies on surgery to preserve the ovaries while removing the uterus. [11–13] Studies using Progestin while preserving the uterus without surgery in Stage IA have also shown good results. [14–16] The Endometrial Cancer Conservative Treatment (E.C.Co.) project endorsed by Gynecologic Cancer Inter-Group (GCIG) reported the promising effect of hysteroscopic resection of endometrial tumor followed by levonorgestrel intrauterine device (LNG-IUD). [17–19] However, the management becomes quite difficult when recurrence occur. Therefore, prophylactic adjuvant treatment should be added to the standard treatment for patients at risk of recurrence. However, aggressive treatment of all patients is unnecessary, incurs socio-economic costs, and adversely affects the patient’s quality of life. In that respect, this study can be considered a very necessary study.

Recent trends in diagnosing and treating endometrial cancer are attempting to create molecular classification and treatment customization. [20, 21] The importance is emerging from the work-up process. However, applying the molecular approach to clinical practice is not yet practical. In terms of cost and treatment efficiency, no evidence shows that the molecular approach increases early diagnosis or improves treatment outcomes. Of course, it will be a good diagnostic tool if it continues to develop in the future, but at this point, we wanted to find a way to treat patients using classic factors. There appears to be an increased risk of regional and distant metastases in the presence of parametrial invasion. The GOG-258 trial [6] showed that systemic chemotherapy plus CCRT was not superior to systemic treatment alone. However, prognosis was not studied by sub-categorizing patients by relapse site. According to our criteria, in stage III patients with parametrial invasion, systemic chemotherapy followed by CCRT may help lower regional recurrence. In the case of FIGO high-grade endometrial cancer, the risk of local and regional metastases increased significantly, but not the risk of distant metastasis. If there is no risk other than a high FIGO grade, it is suggested that CCRT or regional radiotherapy could be considered. LND appears associated with the risk of regional and distant metastases; so, in the high risk group, LND should be performed for accurate staging [22].

It is necessary to note that we only considered the main effects in our multivariate model. That important interactions may have been missed, may have introduced a bias in the model. However, interactions complicate models, and sometimes it is not feasible to evaluate all interaction effects. Furthermore, it is common practice to consider only the main effects in a model when there are many potential factors. For these reasons, we consider our resulting model to be an approximation of the true model. In addition, we created a model that predicts the risk of recurrence based on these results. When focusing on the recall, there was a change in the degree of prediction, and an unexpected recurrence occurred even when both were considered. The accuracy of the model can be further improved through studies with larger sample size. Using the model to predict the probability of recurrence in the patient will assist in the decision regarding the necessity of adding local and/or systemic adjuvant treatment for endometrial cancer. This study has some limitations. First, although we designed a predictive model for recurrence of endometrial cancer, there was no validation group, and the prediction was not accurate. However, this limitation could be addressed by conducting a larger multicenter study with more accumulation of patients’ data. Second, although molecular level classification has become a trend in endometrial cancer recently, [1, 20, 23] our data are lacking in this regard because this study included patients treated more than 10 years ago. Therefore, more interesting results can be expected from future research, including molecular data. Third, patients number of our data was relatively small, huge 95% CI was observed in some factors, but the CI for the risk ratio did not cross 1. This means that this risk factor showed significant effects to recurrences despite the small sample leading to wide CI. Additionally, the wide CIs is not only due to the small sample, but also due to the characteristics of the model we used. Under the logistic model, the CI of the risk ratio is calculated by taking the exponent of the estimation coefficient, so the right tail of the CI tends to be very amplified. Finally, our multivariate model does not satisfy the `rule of 10,` which is a rule of thumb for how many parameters can be reliably fitted for a given sample size. This rule suggests that 10 events may be needed for the smallest category of an outcome, per parameter, in a logistic regression. [24] In our model, 12 predictors were evaluated with relatively low event sizes. Although our model is not perfect, we found that the results of previous studies [25–29] are consistent with those of our binomial logistic model overall, as shown in Table 3. Further, our study extended the analysis in a multinomial logistic model. Therefore, we believe that our multivariate model provides useful insights.

## Data Availability

The datasets used and/or analyzed during the current study are available from the corresponding author on reasonable request.
